# Neurophysiological Profiles in a Family with Multiple SHANK3-Related Phelan–McDermid Syndrome Cases

**DOI:** 10.3390/ijms27031567

**Published:** 2026-02-05

**Authors:** Anastasia Neklyudova, Katerina Lind, Galina Portnova, Ksenia Golovina, Maria I. Mitina, Andrey D. Manakhov, Olga Sysoeva

**Affiliations:** 1 Laboratory of Human Higher Nervous Activity, Institute of Higher Nervous Activity and Neurophysiology, Russian Academy of Science, Moscow 117485, Russia; dr.g.portnova@gmail.com (G.P.); olga.v.sysoeva@gmail.com (O.S.); 2 Institute of Physics and Technology, Ural Federal University, Yekaterinburg 620062, Russia; katerina.lind@gmail.com; 3 Center for Cognitive Science, Sirius University of Science and Technology, Sirius 354340, Russia; zefeni@yandex.ru; 4 Division of Genetics, Sirius University of Science and Technology, Sirius 354340, Russia; maria.geneticlab23@gmail.com (M.I.M.); manakov@rogaevlab.ru (A.D.M.)

**Keywords:** Phelan–McDermid syndrome, *SHANK3* gene, auditory steady-state response, biomarkers, EEG

## Abstract

We present a family case study of Phelan–McDermid syndrome (PMS), a neurodevelopmental disorder caused by haploinsufficiency of the *SHANK3* gene, in which two of three siblings were clinically diagnosed with PMS. Sanger sequencing identified a novel heterozygous deletion in exon 20 of SHANK3 (c.3679del, p.Ala1227Profs*168), predicted to introduce a premature stop codon and truncate the protein; this variant was absent in the unaffected sibling. Auditory steady-state responses (ASSRs) were recorded at 16, 27, and 40 Hz. The 40 Hz ASSR was markedly reduced in both affected siblings, reaching statistical significance in the younger child and remaining non-significant in the older sibling, while it was preserved in the unaffected sibling. These findings suggest that the 40 Hz ASSR is particularly sensitive to SHANK3-related cortical inhibitory dysfunction during childhood and adolescence, with reduced sensitivity in early adulthood. The results highlight the potential of the 40 Hz ASSR as an electrophysiological biomarker in PMS and underscore the need for age-stratified normative control datasets to enable robust individual-level interpretation and support its use in biomarker development, clinical trial stratification, and monitoring of treatment response.

## 1. Introduction

Phelan–McDermid syndrome (PMS), also referred to as 22q13 deletion syndrome, is a neurodevelopmental condition associated with low muscle tone (hypotonia), generalized developmental delays including intellectual disability, limited or absent expressive language abilities, and distinct facial features [1,2]. Estimates suggest that PMS occurs approximately in 1 in 8000–15,000 individuals. Autism spectrum disorder (ASD) is frequently associated with PMS, with reported prevalence rates ranging from 50% to 75% [3,4]. Moreover, the likelihood of an ASD diagnosis in individuals with PMS appears to increase with age, rising from around 19% in childhood to approximately 50% in adulthood [3]. It has also been proposed that PMS may account for about 1% of all ASD [1].

PMS arises from alterations in the 22q13 chromosomal region, with haploinsufficiency of the *SHANK3* gene recognized as the primary pathogenic mechanism underlying the syndrome [3]. PMS follows an autosomal dominant inheritance pattern and is most commonly the result of a de novo genetic variant [5]. The likelihood of recurrence in relatives depends on the specific genetic mechanism responsible for PMS in the affected individual and on the genetic findings in the parents [5]. There is evidence that larger deletions in this region are associated with more severe clinical phenotypes [6,7]. The SHANK3 protein, which shares its name with the gene, functions as a postsynaptic scaffolding molecule crucial for organizing glutamatergic synapses, interacting with key receptor types such as NMDA, AMPA, and metabotropic glutamate receptors [8,9,10,11]. Studies using SHANK3 knockout mouse models have revealed dysfunction in parvalbumin-expressing (PV+) GABAergic interneurons, implicating these inhibitory neurons in the pathophysiology of PMS [12]. Altered function of NMDA receptors disrupts the balance between excitatory and inhibitory signaling in neural circuits, a mechanism linked to autism-like behaviors both in individuals with ASD and in corresponding animal models [13]. Notably, SHANK3 variants are detected in 0.5% of individuals diagnosed with ASD, positioning it as one of the strongest candidate genes for autism [14].

Individuals with PMS face an elevated risk of seizures, with prevalence estimates ranging from 40% to 63% [15,16]. In addition to clinical seizures, many patients exhibit prominent electroencephalographic (EEG) abnormalities, including frequent epileptiform discharges, disorganized cortical activity, and generalized background slowing [17,18]. Spectral EEG analyses further reveal characteristic features such as globally slowed rhythms and diminished occipital alpha dominance [17,19].

Evoked brain responses to sensory stimulation have also been reported to be diminished in PMS. In the visual domain, early event-related potential (ERP) components show reduced amplitudes and P60–N75 amplitude negatively correlated with the size of the chromosomal deletion [20]. In the auditory modality, reductions in the amplitude of the P50 response to repeated tones have been observed [7,21], alongside enhanced habituation of the P2 component, suggesting possible alterations in synaptic plasticity mechanisms [21]. Moreover, individuals with PMS exhibit weaker neural coordination, reflected in decreased phase-locking (ITPC) in the high-frequency alpha–beta and beta–gamma ranges, as well as reduced high-frequency brain activity during tone presentation [7]. This reduction, particularly pronounced in individuals with larger deletions who show less gamma power, may underlie sensory hyposensitivity [7].

Another auditory evoked response that is altered in PMS is the auditory steady-state response (ASSR) that occurs when rhythmic stimulation is presented. ASSR is mostly pronounced at 30–40 Hz [22]. ASSR was reported to be diminished in a group of children with PMS [23] as well as in a patient with SHANK3 duplication [24]. This response is associated with activity of NMDA-receptors on PV+ interneurons, as blockage of these receptors with its antagonist, ketamine, significantly reduces 40 Hz ASSR in mice [25,26], macaques [27] and humans [28]. Moreover, the GABAA receptor–agonist lorazepam increased the amplitude of the 40 Hz ASSR [29]. This makes ASSR a potential biomarker of NMDA receptor hypofunction and PV+ interneuron disruption, which is relevant to syndromes such as PMS as well as broader groups of neurodevelopmental disorders, including ASD and schizophrenia.

In this article, we present a case study of a family in which two of three children exhibit developmental impairments and have been diagnosed with PMS. Genetic examination of the parents and all children revealed pathogenic variants of SHANK3 only in the affected children. Electrophysiological assessment (EEG) was performed in the affected children and their unaffected sibling to investigate neural correlates of the disorder. We specifically focused on ASSR, as it may serve as a potential biomarker of PV+ interneuron dysfunction.

## 2. Results

### 2.1. Phenotyping and Clinical Description

Two sisters of Russian origin from a non-consanguineous family presented with a familial form of Phelan–McDermid syndrome (PMS). The parents and one sibling were clinically unaffected. All three siblings underwent comprehensive neurobiological evaluation at the Laboratory of Human Higher Nervous Activity, Institute of Higher Nervous Activity and Neurophysiology, and genetic testing at Sirius University of Science and Technology.

#### 2.1.1. SH011

SH011 (female) was 24.9 years of age at the time of assessment. She was born as the first child, no complications during pregnancy and delivery were reported.

##### Developmental History

Motor development was markedly delayed. Independent sitting was achieved at 11 months, and independent ambulation was not attained until 3 years of age. These delays were evident from early infancy and remained a prominent feature throughout the developmental trajectory.

Language development followed an atypical course. First words emerged at approximately 10 months, followed by a brief period of normal language acquisition. However, a complete loss of verbal skills occurred by 1.5 years of age. Partial recovery of expressive abilities was observed in late childhood, but this was subsequently followed by a second major regression at 11.5 years.

Psycho-emotional development was significantly impaired. The proband presented with profound intellectual disability and clinical features consistent with autism spectrum disorder (ASD). Repetitive behaviors, including hand clasping and mouthing of objects, were prominent and tended to intensify in response to environmental changes. Her score on the Social Responsiveness Scale (SRS-2) was 103 (social awareness: 7, social cognition: 13, social communication: 37, social motivation: 23, and restricted and repetitive behavior: 23) [30]. This total score falls within the severe range, indicating a high level of social-communication impairment and autistic symptomatology. The profile shows particularly elevated difficulties in social communication and restricted/repetitive behaviors, consistent with her clinical presentation of autism spectrum disorder. Mood lability was also observed; however, episodes of dysregulation were not accompanied by aggression or depressive affect.

##### Clinical Examination

General physical examination revealed a body mass index (BMI) of 27, placing the patient in the overweight range. Neurological examination showed a high pain threshold, with self-scratching performed without signs of distress. Examination of the cranial nerves revealed minimal abnormalities. A mild dysfunction of conjugate eye movements was noted. Thermoregulatory instability was present, characterized by poor heat and cold tolerance and distal hyperhidrosis. Muscle tone was abnormal. Hearing was within normal limits, with no evidence of increased auditory sensitivity or hyperacusis. Sleep disturbances were present and marked by severe insomnia during the regression period, lasting approximately three months. Pubertal development was precocious, with menarche occurring at 8.5 years of age.

At the time of assessment, the proband was receiving pharmacological treatment with trihexyphenidyl (6 mg), levomepromazine (125 mg), thioridazine (75 mg), and biperiden (10 mg).

#### 2.1.2. SH006

SH006 (female) was 11.36 years of age at the time of assessment. She was the middle child in the family, born 13 years after her older sister, SH011. No complications during pregnancy and delivery were reported.

##### Developmental History

Motor development was delayed, with independent sitting achieved at 12 months and walking at 2.5 years. By 8.5 years, she was able to walk long distances and ride a scooter; however, these skills were lost following a regressive episode. At the time of assessment, her gait was slow, and she fatigued rapidly. Stereotyped behaviors observed included hair pulling, mouthing of the hands, and rhythmic foot tapping.

Language development showed early onset, with speech emerging at 10 months, followed by complete regression by 1.5 years. The proband initially demonstrated the ability to understand basic verbal instructions; however, at the time of assessment, responses were inconsistent and delayed.

Psycho-emotional development was markedly impaired, characterized by severe intellectual disability. Following developmental regression, the proband lost previously acquired skills, including drawing, counting, and symbolic play. Autistic features were also present. Repetitive behaviors, such as hand clasping and mouthing of objects, were prominent and tended to intensify in response to environmental changes. Her score on the Social Responsiveness Scale (SRS-2) was 150 (social awareness: 21, social cognition: 26, social communication: 47, social motivation: 21, and restricted and repetitive behavior: 35) [30], consistent with severe social-communication impairment. She exhibited high behavioral reactivity to environmental changes, manifested by prolonged agitation and shouting; these episodes were not associated with aggression or depressive affect.

##### Clinical Examination

General physical examination revealed a body mass index (BMI) of 19, within the normal range. Neurological examination demonstrated mild generalized hypotonia and mild right-sided hemiparesis, as well as neurogenic torticollis. Autonomic dysregulation was present, characterized by poor heat and cold tolerance and distal hyperhidrosis. The proband exhibited a high pain threshold, resulting in self-injurious behaviors such as mouthing, which had led to partial resorption of deciduous teeth. Sleep disturbances were noted, with recent onset of nocturnal wandering and insomnia. No signs of menarche were observed.

At the time of assessment, the proband was receiving pharmacological treatment with thioridazine (25 mg) and alimemazine (5 mg).

##### History of Oncology

Oncological history revealed that the proband had been under specialist follow-up since the age of two due to a diagnosis of lymphatic malformation involving the oropharynx, supraglottic laryngeal structures, and the tongue base. At the time of assessment, she continued to be monitored by an oncologist, with ongoing surveillance and management as clinically indicated.

#### 2.1.3. D125

D125 (female) was 10.95 years of age at the time of assessment. She was the third child in the family, born 14 years after her older sister, SH011. Pregnancy and delivery were uncomplicated, with no reported perinatal difficulties.

##### Developmental History

Developmental milestones were achieved within the normal range, and cognitive functioning was age-appropriate. No neurological or behavioral abnormalities were observed, consistent with typical development. Her score on the Social Responsiveness Scale (SRS-2) was 49 (social awareness: 7, social cognition: 11, social communication: 17, social motivation: 7, and restricted and repetitive behavior: 7) [30], which falls within the typical range and indicates the absence of clinically significant social-communication difficulties or autistic traits.

##### Clinical Examination

General physical examination revealed a body mass index (BMI) of 20, within the normal range. Neurological examination showed no abnormalities. No sleep disturbances were reported. Pubertal development was prepubertal, with no signs of menarche. At the time of assessment, the proband was not receiving any pharmacological treatment.

##### Summary of Phenotypic Features

Both affected siblings present with the core features of PMS: global developmental delay, severe-to-profound intellectual disability, absent expressive language, autistic traits, episodic regression, hypotonia, dysautonomia, hyperphagia, and sleep disturbance [31].

### 2.2. Genetics

Provided by the probands’ parent whole-exome sequencing results for one of the affected girls (SH006) showed a heterozygous single-nucleotide deletion in SHANK3 (NM_033517.1: c.3679delG; p.Ala1227Profs168*). The variant is located in exon 20, within a poly-guanine (poly-G) tract in the region encoding the proline-rich domain (PRD) of SHANK3. The domain mediates crucial interactions with postsynaptic scaffolding proteins, such as Homer and Cortactin. A frameshift initiated by the deletion of a single guanine at codon 1227, substitutes the original alanine with a proline (p.Ala1227Profs168*), which results in the aberrant sequence of 168 amino acids until it is prematurely terminated by a stop codon (p.Ala1227Profs168*).

#### 2.2.1. Segregation Analysis

**Figure 1:**
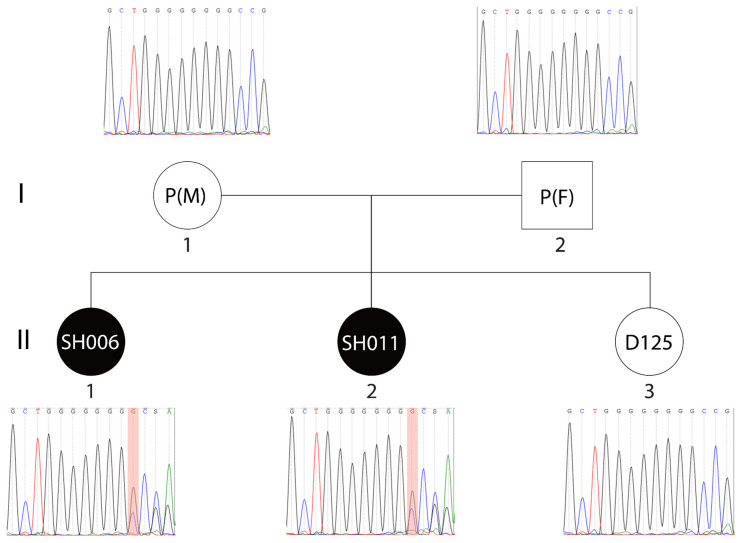
Segregation analysis of the SHANK3 c.3679del variant. Sanger sequencing chromatograms demonstrate the heterozygous deletion in both probands (SH006, SH011) and the reference sequence in the parents and unaffected sister (D125). Nucleotide peaks are colour-coded according to standard Sanger convention: adenine (A) in green, cytosine (C) in blue, guanine (G) in black, and thymine (T) in red. The variant position is highlighted by a shaded vertical region in the chromatograms.

Sanger sequencing identified the c.3679delG variant in both affected siblings but not in their unaffected sister (D125) or either parent (see Figure 1). This pattern of inheritance demonstrates that the variant co-segregates perfectly with the syndrome phenotype within the sibling cohort. The fact that neither parent carries the variant suggests either a de novo origin of the variant or more likely a gonadosomatic mosaicism with a low-level variant fraction in somatic tissues in one of the parents, which explains its recurrence in the children.

#### 2.2.2. In Silico Modeling and Predicted Molecular Effect

**Figure 2:**
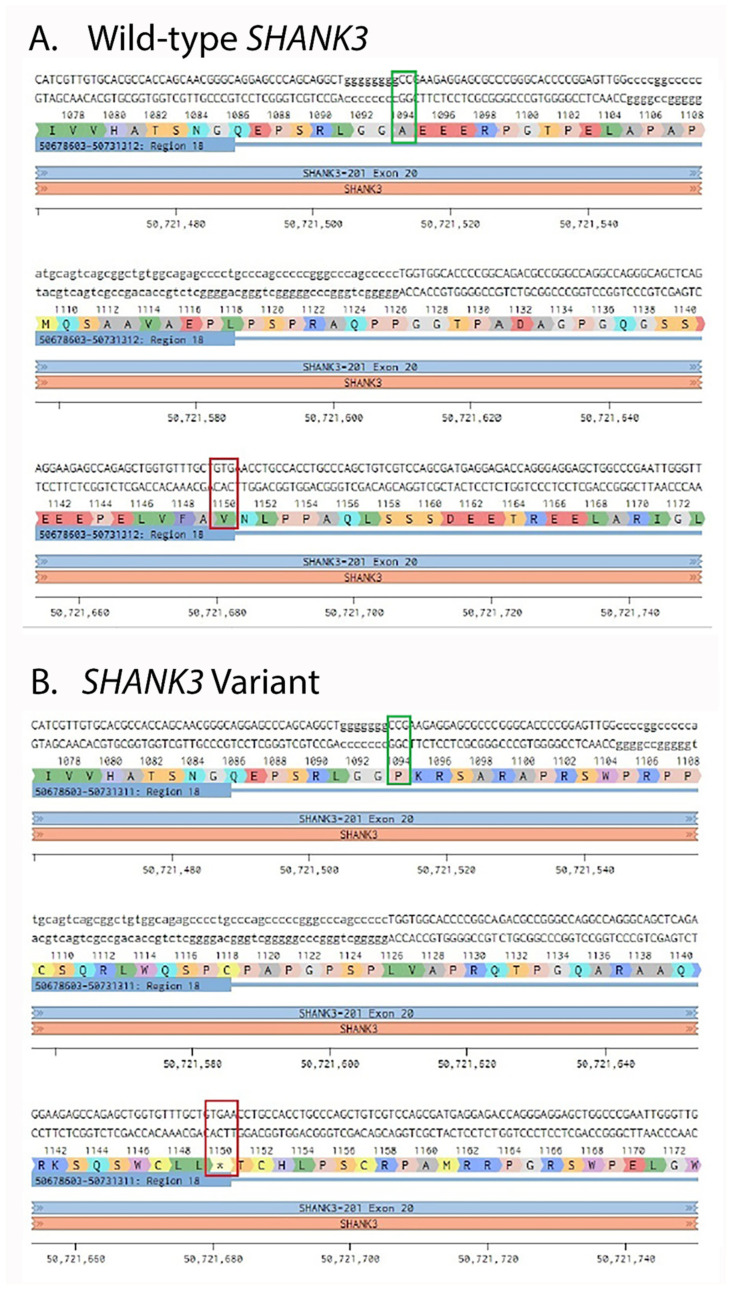
In silico visualization of the SHANK3 c.3679delG (p.Ala1227Profs*168) variant. (**A**) Wild-type SHANK3 sequence showing the poly-G region in exon 20. (**B**) Mutant sequence after deletion of a single guanine (c.3679delG), leading to a frameshift and the appearance of a premature stop codon. The green box marks the deleted guanine within the poly-G tract, and the red box indicates the position of the newly generated stop codon. Capital letters in the amino-acid track denote the single-letter amino-acid code. Amino acids are colour-coded according to their physicochemical properties (as defined by Benchling): hydrophobic residues (e.g. A, V, L, I, F, W, M) are shown in green, polar uncharged residues (e.g. S, T, N, Q) in blue, positively charged residues (e.g. K, R) in purple, negatively charged residues (e.g. D, E) in red, and special cases (e.g. P, G, C) in distinct contrasting colours.

In silico analysis using the Benchling platform predicted that the c.3679delG variant causes a frameshift, resulting in a truncated protein product (p.Ala1227Profs*168) that lacks the C-terminal Homer-binding and SAM domains. This truncation leads to severe structural disruption, including a predicted loss of α-helical content that would prevent multimeric scaffolding, thereby impairing postsynaptic signaling and organization. The premature stop codon is expected to trigger nonsense-mediated mRNA decay (NMD), resulting in a loss-of-function (LoF) effect (Figure 2).

According to the American College of Medical Genetics and Genomics and the Association for Molecular Pathology guidelines [32], the SHANK3 c.3679delG (p.Ala1227Profs168)* variant meets the following evidence categories: PVS1, PM1, PM2, PP1, PP4, therefore supports the classification of this variant as Pathogenic (evidence code and criteria are presented in Table A1).

### 2.3. Electroencephalography

#### 2.3.1. Clinical EEG Examination

**Figure 3:**
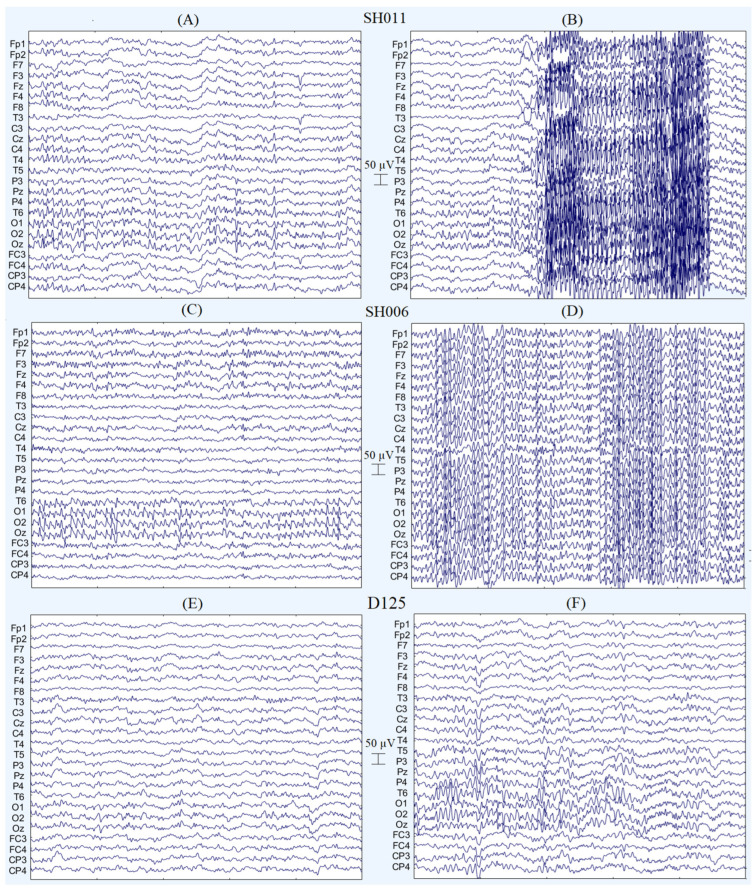
Examples of clinical EEG for participants SH011, SH006 and D125 with duration 5 s for each image. SH011: (**A**,**B**)—resting state condition with eyes opened; (**A**)—fragment free of pseudogeneralization; (**B**): hypersynchronized 14 Hz activity dominated over right hemisphere regions. SH006: (**C**,**D**) resting state condition with eyes opened; (**C**)—fragment sharp-wave 12 Hz activity dominate in occipital areas; (**D**): hypersynchronized 12 Hz activity dominated over all regions. D125: (**E**)—resting state condition with eyes opened; (**F**)—resting state condition with eyes closed.

The clinical EEG assessment of SH011 revealed disorganized background activity with diffuse slow waves at 3–5 Hz and irregular 6–8 Hz rhythms of low index and atypical topography. Alpha activity was poorly expressed, appearing only as isolated waves, while beta activity (22–28 Hz, up to 40 µV) predominated with eyes open. Photic stimulation further increased disorganization and provoked paroxysmal activity. Paroxysmal and epileptiform discharges were represented by hypersynchronized beta rhythm at 14 Hz (14.2–15.1 Hz), reaching amplitudes up to 130 µV, with a duration of up to 5 s and an activity index of ~10% and domination at the right hemisphere. No significant interhemispheric asymmetry or focal abnormalities were observed. Examples of these events are presented in Figure 3B.

The clinical EEG assessment of SH006 revealed diffuse low-amplitude slow-wave activity at 1.5–2.5 Hz with amplitudes up to 20 µV. With eyes open, an occipital alpha rhythm at 11–13 Hz and amplitudes reaching 100 µV was observed, showing a tendency toward generalization. The visual and sensorimotor resting rhythms displayed variable frequencies within the same range, with somewhat blurred topography and signs of hypersynchronization. Beta activity (14–28 Hz, up to 20 µV) predominated with eyes open. Background activity was generally disorganized, and no significant interhemispheric asymmetry was detected. Paroxysmal episodes of synchronous generalized alpha activity at 12 Hz were recorded, arising and terminating abruptly, with a duration of approximately 3 s. Sporadic epileptiform discharges were noted in the left hemisphere. Examples of these events are presented in Figure 3A.

The clinical EEG assessment of D125 showed that the posterior dominant rhythm is well-formed and appropriate at (9–11 Hz with amplitudes up to 95 µV) for the patient’s age. The posterior dominant rhythm was reactive, showing normal suppression (~60% amplitude reduction) with eye opening. The background activity is symmetric and shows normal organization during both wakefulness. There was no evidence of focal or generalized epileptiform activity.

#### 2.3.2. Auditory Steady-State Response

For the study of auditory steady-state response (ASSR), we additionally recorded EEG from 30 typically developing children, with three age-matched control groups (10 children each) for SH006, SH011, and D125. This was necessary because the ASSR, particularly at 40 Hz, shows a pronounced developmental increase, with a peak during adolescence, probably indicating the development of the GABAergic system [33]. The groups had mean ages of 11.67 ± 0.55 years (range: 10.82–12.44; control for SH006, 11.36 years), 23.45 ± 2.44 years (19.6–27.02; control for SH011, 24.9 years), and 10.55 ± 0.36 years (9.99–11.15; control for D125, 10.95 years).

**Figure 4:**
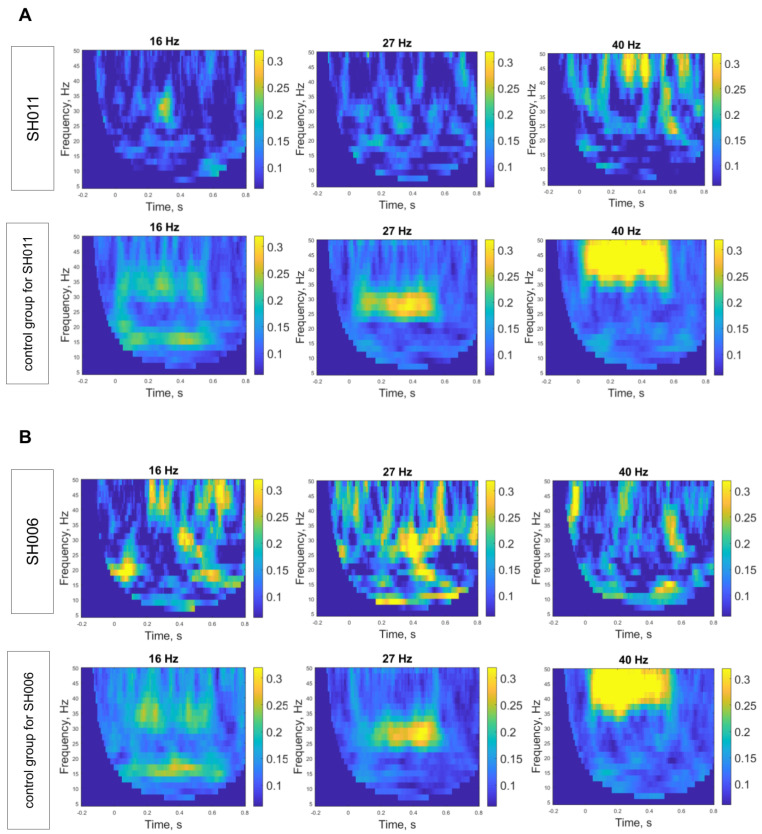

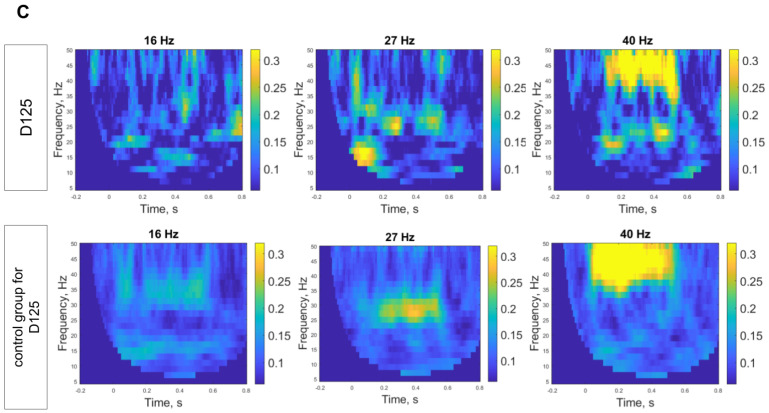
Time frequency representation of Intertrial phase coherence (ITPC) at different frequencies for SH011 (**A**), SH011 (**B**), D125 (**C**) and corresponding control group.

All children listened to 500 ms click trains with interstimulus intervals of 500–800 ms. Three sessions were conducted, differing in click train repetition rates: 16, 27, and 40 Hz. ITPC values for each stimulation frequency for SH011, SH006, and D125 are presented in Table 1, and graphical representations of the ASSRs for patients, their unaffected sibling and control groups are shown in Figure 4.

To compare the single patient’s score with the control sample (*n* = 10) statistically, we used the Crawford and Howell modified t-test and its Bayesian extension [34,35,36], which estimate standardized case scores and the proportion of the normative population expected to score below the case.

**Figure 5:**
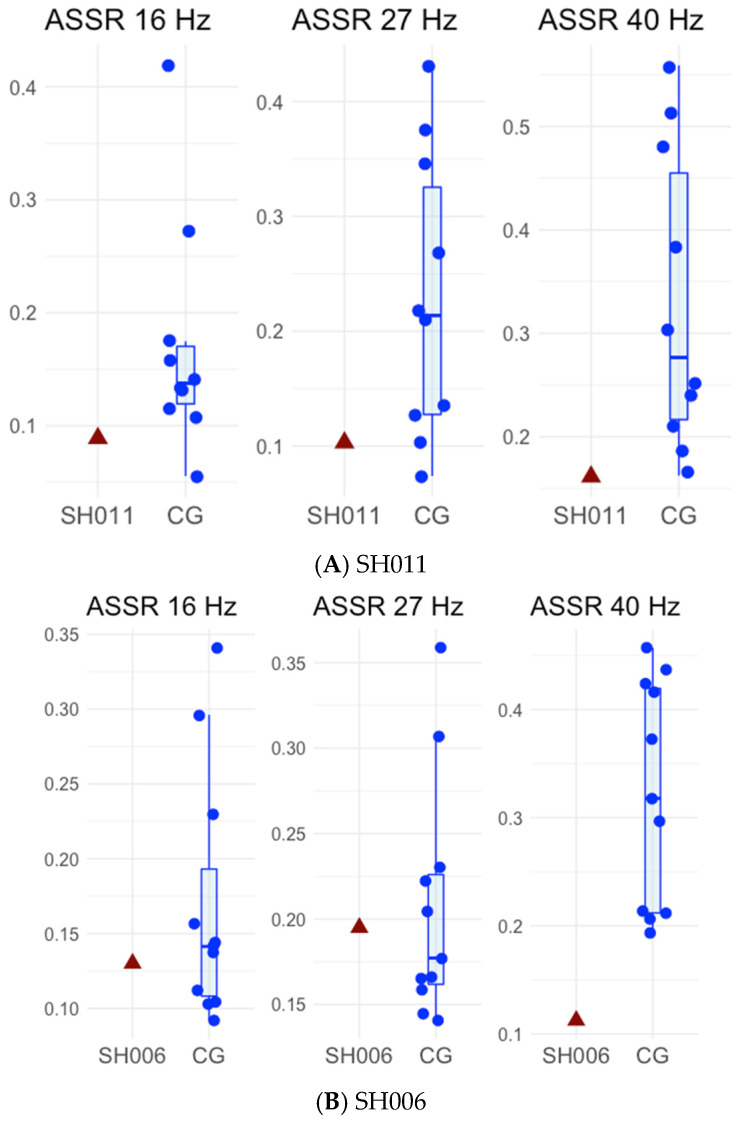

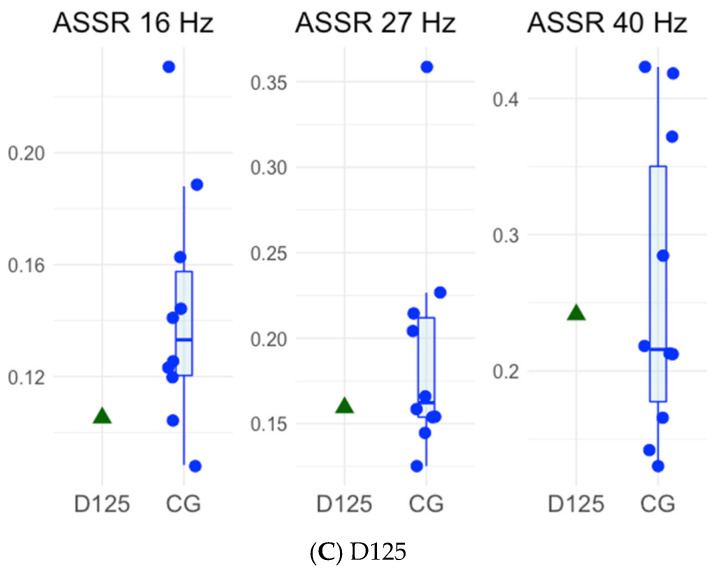
Comparison of ASSR at 16, 27, and 40 Hz for patients ((**A**): SH011, (**B**): SH006), their unaffected sibling ((**C**): D125), and the corresponding control groups (CG).

For SH011, Bayesian single-case comparisons indicated that at 16 Hz the ASSR value (0.09) did not differ reliably from the control mean (M = 0.17, SD = 0.10; *p* = 0.23), yielding a standardized score of −0.79 (95% credible interval [−1.47, −0.06]) and placing the case below approximately 79% of the control population (95% CI [52.3%, 92.9%]). At 27 Hz, the ASSR value (0.10) remained lower than the control mean (M = 0.23, SD = 0.12; *p* = 0.18), yielding a standardized score of −1.01 (95% credible interval [−1.73, −0.23]) and placing the case below approximately 82% of controls (95% CI [59.0%, 95.8%]). At 40 Hz, the ASSR value (0.16) was lower than the control mean (M = 0.33, SD = 0.14; *p* = 0.14), yielding a standardized score of −1.16 (95% credible interval [−1.93, −0.41]) and placing the case below approximately 85% of controls (95% CI [66.0%, 97.3%]). Although SH011′s ASSR values did not significantly differ from those of the control group across frequencies, likely reflecting limited statistical power due to the small control sample, the largest estimated deficit was observed at 40 Hz. Results are presented in Figure 4A and Figure 5A.

For SH006, Bayesian single-case comparisons indicated that at 16 Hz the ASSR value (0.13) did not differ reliably from the control mean (M = 0.18, SD = 0.10; *p* = 0.33), yielding a standardized score of −0.49 (95% credible interval [−1.15, 0.19]) and placing the case below approximately 67% of the control population (95% CI [42.5%, 87.4%]). At 27 Hz, the ASSR value (0.195) again did not differ from controls (M = 0.21, SD = 0.06; *p* = 0.42), yielding a standardized score of −0.22 (95% credible interval [−0.84, 0.42]) and placing the case below approximately 58% of controls (95% CI [33.9%, 79.9%]). At 40 Hz, the ASSR value (0.113) was significantly lower than the control mean (M = 0.32, SD = 0.11, *p* = 0.04), yielding a standardized score of −1.83 (95% credible interval [−2.86, −0.78]) and placing the case below approximately 94% of controls (95% CI [78.2%, 99.8%]). Results are presented in Figure 4B and Figure 5B.

For D125, Bayesian single-case comparisons indicated that at 16 Hz the ASSR value (0.105) did not differ reliably from the control mean (M = 0.14, SD = 0.04; *p* = 0.21), yielding a standardized score of −0.89 (95% credible interval [−1.61, −0.14]) and placing the case below approximately 79% of the control population. At 27 Hz, the ASSR value (0.159) was also similar to controls (M = 0.19, SD = 0.07; *p* = 0.33), yielding a standardized score of −0.46 (95% credible interval [−1.10, 0.20]) and placing the case below approximately 67% of controls. At 40 Hz, the ASSR value (0.241) was close to the control mean (M = 0.26, SD = 0.11; *p* = 0.44), yielding a standardized score of −0.15 (95% credible interval [−0.77, 0.48]) and placing the case below approximately 56% of controls. Results are presented in Figure 4C and Figure 5C.

In summary, across the three participants, ASSRs were generally comparable to controls at 16 and 27 Hz. The largest reductions were observed at 40 Hz for SH006, reaching statistical significance, and for SH011, which, however, did not reach significance. The unaffected sibling D125 showed no notable reductions at any frequency, including 40 Hz.

## 3. Discussion

Our report describes a family with two children with Phelan–McDermid syndrome (PMS) carrying a pathogenic mutation in SHANK3 (SH011 and SH006) and their unaffected sibling (D125). To our knowledge, this is the first description of the neurophysiological phenotype in siblings discordant for PMS within the same family.

We identified a heterozygous frameshift variant in the *SHANK3* gene (NM_033517.1: c.3679delG; p.Ala1227Profs*168) in both affected siblings, arising within a homopolymeric poly-G tract in exon 20 that encodes a glycine-rich segment of the proline-rich domain (PRD). Single-base slippage in such tracts is a recognized mutational mechanism and explains the recurrent indel hotspot at this locus [37]. A previous study reported frameshift genetic variants in this site, although in contrast with our findings, they identified a heterozygous duplication, c.3679dupG (p.Ala1227Glyfs69)*, which produces an equivalent frameshift and premature stop codon [38]. The recurrent c.3679dupG variant was identified in three unrelated individuals diagnosed with PMD syndrome and ASD, further supporting the high instability of this sequence motif and its pathogenic relevance in SHANK3-related neurodevelopmental disorders [38].

Similar small insertion and deletion events in the poly-G tract of SHANK3 have been repeatedly described in individuals with ASD [39]. The most common is the heterozygous c.3679dupG (p.Ala1227Glyfs69), observed in 18 cases from 16 families, whereas a rarer deletion c.3679delG (p.Ala1227Profs57) has been identified in a single female proband presented with ASD, but was not diagnosed with PMS. Both events arise within the same homopolymeric guanine stretch, supporting a shared mutational mechanism and functional equivalence that can lead to either ASD or PMS–like phenotypes described in the present case report. At the time of writing, we were unable to locate this variant in major genomic databases, including OMIM, ClinVar, and gnomAD.

Epilepsy is highly prevalent among patients with PMS. A meta-analysis across 14 studies estimated the pooled prevalence of seizures at 32% [19], while lifetime prevalence in adults can reach up to 60% [40]. Consistent with these observations, the clinical EEG assessments of SH011 and SH006 revealed altered rhythms, including diffuse slow-wave activity, disorganized background, and abnormal beta and alpha patterns, accompanied by epileptiform activity. In contrast, the unaffected sibling D125 exhibited well-formed, age-appropriate posterior rhythms with normal organization and no epileptiform discharges, highlighting the specificity of cortical abnormalities in the affected patients. None of the affected siblings were receiving anticonvulsant medication; their primary pharmacological treatment consisted of neuroleptics. Therefore, the observed EEG abnormalities cannot be attributed to antiseizure therapy [41]. The paroxysmal activity detected in both probands appears intrinsic to their neurophysiological profile rather than medication-related and likely reflects a characteristic feature of their pathological EEG activity.

### 40 Hz ASSR as an Early Biomarker of SHANK3 Disruption

The main focus of our investigation was the auditory steady-state response (ASSR), a well-established marker of NMDA-receptors and PV+ interneurons dysfunction [25,42], both of which depend on SHANK3 activity [43,44]. In our study, ASSRs were preserved in the unaffected sibling D125. In both patients, the largest reductions were observed at 40 Hz, with SH011 showing the most pronounced deficit and SH006 exhibiting a significant reduction at the same frequency.

Alterations in the 40 Hz ASSR have been previously described in an individual with a SHANK3 microduplication and in a cohort of children diagnosed with PMS [23,24]. Previous studies also reported impairments in resting-state gamma-band activity in children with PMS [7,45]. Moreover, similar reduction in 40 Hz ASSR was observed in children with Rett syndrome [46], another genetic syndrome that causes neurodevelopmental delay and is associated with PV+ interneurons dysfunction [47,48,49]. Together, these findings indicate that reduced 40 Hz ASSR is a recurring feature across several neurodevelopmental syndromes involving SHANK3 dysfunction or impaired PV+ interneuron activity, highlighting its potential as a transdiagnostic biomarker.

The amplitude of ASSR at 40 Hz demonstrates a pronounced developmental trajectory: it increases from childhood, peaks during adolescence, and subsequently declines in early adulthood [33,50]. Interestingly, this U-shaped developmental trajectory was observed only for 40 Hz stimulation, but not for 30 Hz or 20 Hz [33]. This may explain why the deficit in SH006, the younger sibling (11.6 years old), reached significance despite the small control sample, whereas the same sample size was insufficient to detect a significant effect in the older sibling, SH011 (24.9 years old), who nonetheless exhibited a pronounced deficit.

The developmental trajectory of 40 Hz ASSR is thought to reflect the interplay of maturational processes, including the strengthening of fast GABAergic inhibition [51,52], which promotes gamma synchronization, and synaptic pruning, which reduces excessive gamma activity [53,54]. Disruption of these processes may explain why children with SHANK3 mutations show a reduction in the 40 Hz ASSR. Such alterations may reflect impaired maturation of inhibitory circuits and excitatory/inhibitory balance, contributing to broader developmental delays observed in PMS. Together, these findings suggest that the 40 Hz ASSR may serve as a sensitive biomarker of cortical maturation and functional disruption in childhood and adolescence, but may be less informative in young adulthood.

Behaviorally, ASSR is associated with speech-in-noise perception [55] and with click rate discrimination [56]. The impairments in these processes may have a cascading effect on speech development. In children with Rett syndrome, reductions in the 40 Hz ASSR were associated with delayed or impaired speech development: the decrease was most pronounced in children with absent speech and less marked in those able to use words or simple phrases, highlighting the functional relevance of this neurophysiological measure [46]. Alterations in the 40 Hz ASSR have also been associated with language difficulties in children with ASD [57,58]. No similar data are yet available for PMS, but investigating this relationship in future studies would be of considerable interest. Taken together, these findings suggest that the 40 Hz ASSR may serve as a potential marker of psychophysiological deficits associated with disruptions in the *SHANK3* gene, as well as other genes affecting PV+ interneurons and NMDA receptor function.

## 4. Methods and Materials

All methods were carried out in accordance with relevant guidelines and regulations for laboratory work. The study was approved by the local ethics committee of the Institute of Higher Nervous Activity and Neurophysiology, Russian Academy of Sciences, protocol number 3, date of approval 10 July 2020, and of Sirius University of Sciences and Technology. The study was conducted following the ethical principles regarding human experimentation (Helsinki Declaration).

### 4.1. Clinical Evaluation

Prior to the assessment, we conducted a comprehensive review of all available medical records and a structured interview with the parents to elicit a complete family medical history. The clinical phenotype was examined by a physician specializing in developmental disorders, focusing on dysmorphic features, growth parameters, neurological status, and behavioral presentation of the probands. Furthermore, electroencephalographic (EEG) recordings of all three siblings were reviewed and interpreted by an experienced clinical neurophysiologist, specializing in epilepsy. This clinical evaluation confirmed the PMS diagnosis in the two probands, and validated the unaffected status of the youngest sister and parents, therefore providing the necessary clinical framework for correlating the genetic and neurophysiological findings.

### 4.2. Genetic Examination

#### 4.2.1. Sanger Sequencing and Segregation Analysis

Genomic DNA from the peripheral blood was extracted using QIAamp DNA Mini Kit, following the manufacturer’s protocol (Qiagen, Hilden, North Rhine-Westphalia, Germany). Sanger sequencing was performed to validate the selected mutation (hg38, NC_000022.11:g.50,721,512del). Primers for PCR amplification (F:GACCCCTGTTTGTGGATGTA, R:AGGAGGTGTGGGTGTCAGT) were designed in Primer3 software, and PCR was performed with GenPack PCR Core (Isogen, Moscow, Russia). The resulting amplicons were cleaned with a Cleanup Standard Kit (Evrogen, Moscow, Russia) and processed with Big-Dye Terminator v3.1. Cycle Sequencing Kit (Applied Biosystems, Carlsbad, CA, USA) following the manufacturer’s protocol. Probes were purified using DyeEx 2.0 Spin Kit (Qiagen, North Rhine-Westphalia, Germany) and sequenced on a 3730 DNA Analyzer (Applied Biosystems) at Sirius University of Science and Technology.

Following its confirmation in the proband, the novel variant in the *SHANK3* gene was screened for in all available first-degree relatives (mother, father, and unaffected sister) to analyze co-segregation.

#### 4.2.2. Bioinformatic Tools

Forward and reversed FASTA reads were aligned against the human reference genome (GRCh38.p14) using the Clustal Omega algorithm within the Jalview software (version 2.11.4.1). Sequence chromatograms were visualized using Chromas software (version 2.6.6) to inspect specific nucleotide positions. Samples displaying sequence divergence from the reference were further analyzed by modeling the putative mutation in silico using the Benchling platform to determine its effect on the coding sequence.

To clinically annotate the variant identified by Sanger sequencing, we used several databases. These included the NCBI’s ClinVar, which contains information on the association of genetic variants with human diseases, the Online Mendelian Inheritance in Man (OMIM) database, and the Genome Aggregation Database (gnomAD) for general population frequency analysis. The variant was then classified according to the guidelines established by the American College of Medical Genetics and Genomics (ACMG) and the Association for Molecular Pathology (AMP) [32].

### 4.3. Electroencephalographic Study

#### 4.3.1. Participants

In addition to the studied family members, we recorded EEG from 30 typically developing children, with 10 children matched as control groups for SH006, SH011, and D125, respectively. The first group had a mean age of 11.67 ± 0.55 years (range: 10.82–12.44) and served as the control group for SH006 (11.36 years). The second group had a mean age of 23.45 ± 2.44 years (range: 19.6–27.02) and served as the control group for SH011 (24.9 years). The third group had a mean age of 10.55 ± 0.36 years (range: 9.99–11.15) and served as the control group for D125 (10.95 years). None of the participants from control groups had neurologic or psychiatric disorders according to their parents’ reports. Parents for children from both groups were requested to fill out the Russian-adapted version of the Social Responsiveness Scale for children [30].

Participants were recruited from the advertisement in the local community. The study was approved by the local ethics committee of the Institute of Higher Nervous Activity and Neurophysiology, Russian Academy of Sciences, protocol number 3, date of approval 10 July 2020, and was conducted following the ethical principles regarding human experimentation (Helsinki Declaration). Parents or legal representatives gave written informed consent to the children’s participation in the study. All children provided their verbal consent to participate in the study and were informed about their right to withdraw from the study at any time during the testing.

#### 4.3.2. Stimulation

The sound stimuli, consisting of click trains, were created using custom software within the MATLAB environment version R2025a (The MathWorks, Natick, MA, USA) and presented through on-ear headphones. Each click was a 0.2 ms rectangular pulse, repeated at various frequencies (16 Hz, 27 Hz, and 40 Hz) for a total duration of 500 ms. In each session, 150 click trains were presented at a sound pressure level (SPL) of 65 dB, with interstimulus intervals randomly varying between 500 and 800 ms. The stimuli were delivered using the Presentation software version 25.1 (NeuroBehavioral Systems, Albany, CA, USA). Participants were seated comfortably during the session and were not assigned any specific tasks related to the auditory stimuli. Children watched a silent video of their choice while the experiment was conducted.

#### 4.3.3. EEG Acquisition and Processing

Continuous electroencephalographic (EEG) data were recorded using the NeuroTravel system with 28 scalp electrodes positioned according to the international 10–10 system. Reference electrodes were placed on the earlobes, and the ground electrode was positioned at the midline of the forehead. The EEG signal was sampled at a rate of 500 Hz and later filtered offline with a bandpass filter ranging from 0.016 to 70 Hz. A notch filter at 50 Hz was applied to the raw data to remove line noise, and electrode impedances were maintained below 10 kΩ.

The data was processed using the MATLAB-based toolbox FieldTrip [59] alongside custom scripts. The continuous EEG data were segmented into epochs starting 200 ms before and ending 1000 ms after stimulus onset. Channels with outlier signals were replaced using interpolation, and epochs were excluded from each participant’s dataset if their amplitudes exceeded 3 standard deviations from the mean. This procedure was repeated iteratively until no values exceeded this threshold.

Time–frequency decomposition was performed with Morlet wavelets (7 cycles) using 2 Hz increments. For the stimulation rates of 16, 27, and 40 Hz, the analyzed frequency range was 1–50 Hz. We then conducted an intertrial phase coherence (ITPC) analysis to assess the phase-locked stability across trials. ITPC values range from 0 to 1, with values closer to 1 indicating greater phase coherence.

As can be seen in Figure 4, ITPC values lie wider than the frequency of stimulation. This broader gamma-band phase locking likely reflects a combination of finite-duration stimulation, time–frequency spectral smoothing, and the intrinsic bandwidth of cortical gamma oscillatory networks. Specifically, auditory steady-state responses to a 40 Hz drive often engage a wider low-gamma range (e.g., ~30–50 Hz) rather than producing a strictly narrowband peak, as established in prior literature [22,33,56,57]. Thus, the observed ITPC distribution is consistent with established neurophysiological properties of auditory gamma-band entrainment. However, we focused our attention only on frequencies that were close to the frequencies of stimulation to ensure our conclusions were based on the direct neural response to the stimulus.

ITPC values were averaged over the 0–500 ms time window and within 6 Hz around the stimulation frequency (e.g., 13–19 Hz for 16 Hz, 24–30 Hz for 27 Hz, and 37–43 Hz for 40 Hz stimuli). Statistical analysis was performed on the FCz electrode, where the ASSR is most pronounced.

#### 4.3.4. Clinical EEG Evaluation

Clinical EEG was acquired according to a standard pediatric protocol. The recording comprised resting-state segments with eyes-open and eyes-closed (when it was possible) conditions, followed by standard activation procedures. We also included photic stimulation at frequencies ranging from 1 to 30 Hz, when it was possible.

#### 4.3.5. Statistical Analysis

To compare each single patient’s score with the control sample (*n* = 10), we used the Crawford and Howell modified t-test and its Bayesian extension [34,35]. These methods provide a standardized case score (Z-CC) and estimate the proportion of the normative population expected to score below the case. Bayesian Tests of Deficit were implemented in R using the singcar package, with 10,000 iterations and a 95% credible interval, under a one-tailed hypothesis that the patient’s score would be lower than controls (alternative = “less”). This approach is well suited for single-case analyses with small normative samples, allowing assessment of whether an individual’s performance deviates reliably from a control group while accounting for uncertainty in the estimate of the population parameters.

## 5. Conclusions

This study describes a familial case of Phelan–McDermid syndrome involving two affected siblings carrying an identical heterozygous pathogenic SHANK3 frameshift variant (c.3679delG, p.Ala1227Profs*168), absent in their unaffected sibling and in parental blood DNA, strongly suggesting parental germline mosaicism as the underlying mechanism. The variant, occurring within a homopolymeric G-tract in exon 20, results in a premature termination codon and SHANK3 truncation. The absence of this variant in population databases, its predicted loss-of-function impact, its de novo occurrence in both affected siblings, and its clear segregation with the clinical phenotype collectively support its classification as pathogenic.

This is the first report to provide a neurophysiological comparison within a single family containing siblings discordant for PMS. The key finding is a pronounced and significant deficit of the 40 Hz ASSR in the younger affected child (SH006), with pronounced, however non-significant, deficit of 40 Hz ASSR in the older affected sibling (SH011) and preserved 40 Hz ASSR in the unaffected sibling (D125). Notably, ASSR at 16 and 27 Hz remained unaltered or less decreased. Taken together, the findings suggest that the 40 Hz ASSR may be most informative during childhood and adolescence, with its sensitivity potentially diminishing by early adulthood. This age-dependent variability should be carefully considered in future studies using the 40 Hz ASSR for biomarker development, clinical trial stratification, or monitoring of treatment response in SHANK3-related neurodevelopmental disorders. In addition, the use of the 40 Hz ASSR as a reliable biomarker will require substantially broadened and age-stratified normative control databases, enabling robust interpretation of individual-level deviations across developmental stages.

## Figures and Tables

**Table 1:** ITPC values for each participant averaged within ±3 Hz around the presentation frequency and within the 0–500 ms time window.

	SH011	SH006	D125
16 Hz	0.089	0.131	0.105
27 Hz	0.103	0.195	0.159
40 Hz	0.162	0.113	0.241

## Data Availability

The original contributions presented in this study are included in the article. Further inquiries can be directed to the corresponding authors.

## Abbreviations

The following abbreviations are used in this manuscript:

EEG	Electroencephalography
ITPC	Intertrial phase coherence
ASSR	Auditory steady-state response
ASD	Autistic spectrum disorder
PMS	Phelan–McDermid syndrome

## Appendix A

**Table A1:** Criteria for Classifying the variant as Pathogenic.

Evidence Code	Criterion	Evidence of Pathogenicity
PVS1	Null variant (nonsense, frameshift, canonical +/−1 or 2 splice sites, initiation codon, single or multi-exon deletion) in a gene where loss of function (LOF) is a known mechanism of disease	Very Strong
PM1	Located in a mutational hot spot and/or critical and well-established functional domain (e.g., active site of an enzyme) without benign variation	Moderate
PM2	Absent from controls (or at extremely low frequency if recessive) in Exome Sequencing Project, 1000 Genomes or ExAC	Moderate
PP1	Co-segregation with disease in multiple affected family members in a gene definitively known to cause the disease	Supporting
PP4	Patient’s phenotype or family history is highly specific for a disease with a single genetic etiology	Supporting
